# Postoperative effects and complications of intrathecal morphine compared to epidural analgesia in patients undergoing intracorporeal robot-assisted radical cystectomy: a retrospective study

**DOI:** 10.1186/s12871-023-02141-w

**Published:** 2023-05-22

**Authors:** Sanne de Bock, Carl J. Wijburg, Mark V. Koning

**Affiliations:** 1 Resident Intensive Care Unit, Rijnstate Hospital, Arnhem, The Netherlands; 2 Urologist, Rijnstate Hospital, Arnhem, The Netherlands; 3 Anesthesiologist-Intensivist, Rijnstate Hospital, Arnhem, The Netherlands; 4 Department of Anesthesia and Critical Care, Wagnerlaan 55, 6815 AD Arnhem, The Netherlands

**Keywords:** Intrathecal morphine, Epidural analgesia, Robot assisted radical cystectomy, Opioids

## Abstract

**Background:**

Analgesia after robot assisted radical cystectomy aims to reduce postoperative pain and opioid consumption, while facilitating early mobilization and enteral nutrition and minimizing complications. Epidural analgesia is currently recommended for an open radical cystectomy, but it is unclear if intrathecal morphine is a suiting, less-invasive alternative for a robot-assisted radical cystectomy.

**Methods:**

The analgesic method of choice changed from epidural anesthesia to intrathecal anesthesia for patients undergoing a robot-assisted radical cystectomy. This single-center retrospective study aims to investigate if there is a difference between epidural and intrathecal analgesia in postoperative pain scores, opioid consumption, length of hospital stays and postoperative complications. An Propensity Matched Analysis was added to conventional analysis to consolidate the findings.

**Results:**

The study population consisted of 153 patients of whom 114 received an epidural catheter with bupivacaine/sufentanil and 39 received a single shot of intrathecal bupivacaine/morphine. Mean pain scores on the first two postoperative days (POD) were slightly higher in the intrathecal analgesia group (epidural versus intrathecal analgesia, NRS POD0: 0(0–2)[0–8] versus 1(0–3)[0–5], *p* = 0.050; POD1: 2(1–3)[0–8] versus 3(1–4)[0–7], *p* = 0.058; POD2: 2(0–3)[0–8] versus 3(2–4)[0–7], *p* = 0.010). Total postoperative morphine consumption was similar over the first seven days: 15 mg (5–35)[0–148] in the epidural group versus 11 mg (0–35)[0–148] in the intrathecal morphine group, *p* = 0.167. Length of hospital stay and time until fit for discharge where slightly higher in the epidural group (respectively 7 days (5–9)[4–42] versus 6 days (5–7)[4–38], *p* = 0.006, and 5 days (4–8)[3–30]) versus 5 days (4–6)[3–34], *p* = 0.018). There was no further difference in postoperative course.

**Conclusions:**

This study showed that the effects of epidural analgesia and intrathecal morphine are comparable and that intrathecal morphine may be a suiting alternative for epidural analgesia.

## Background

Robot assisted radical cystectomy (RARC) is considered a minimal invasive form of cystectomy with reduced intra-operative blood loss and shortened hospital stay [1]. Analgesia after a RARC aims to reduce somatic and visceral pain and opioid consumption, while facilitating early mobilization and enteral nutrition and minimizing complications such as ileus, hypotension and pneumonia [2, 3]. Epidural analgesia (EDA) is currently the preferred technique in an open radical cystectomy, because of its superior pain control and reduction of complications when compared to systemic opioids [4]. Even though there may not be much difference in pain between RARC and open radical cystectomy [5], in many other laparoscopic procedures the use of EDA is not as clearly indicated as in open abdominal surgery [6–8]. Therefore, alternatives to EDA for laparoscopic surgery are currently investigated, which has led to a renewed interest in intrathecal hydrophilic opioids [9]. In the RARC procedure, a reasonable alternative to EDA may be intrathecal analgesia, which has a higher success rate of administration and a different profile of side-effects.

Both epidural and intrathecal analgesia block both somatic and visceral pain, but have their own benefits and side-effects. Depending on patient and surgical factors, one analgesic method can be preferred over the other. The benefits of EDA by using a catheter are the adjustable and prolonged duration of analgesia and the superior analgesia provided by a properly placed catheter. The disadvantages of EDA are a reduced success rate, side effects such as hypotension and motor blockade and the delayed possibility to (re)initiate anticoagulants, if needed. Intrathecal analgesia, commonly obtained by morphine (ITM), has a high success rate, important opioid-sparing effects and sparing of motor and sympathetic nerves [9]. The disadvantages are the fixed duration of effect and side-effects such as urinary retention, pruritus and a delayed respiratory depression, the two latter being dose dependent [9]. So far, EDA and ITM have not been compared in a RARC-procedure, while both may be suitable methods of analgesia.

Based on positive studies and local availability, our institution changed from an analgesic regimen with epidural analgesia and paracetamol to an analgesic regimen with intrathecal morphine, supplemented with paracetamol and NSAIDs. The current study aims to retrospectively investigate if there is a difference in postoperative pain scores, opioid consumption, length of hospital stay and postoperative complications between the EDA and ITM cohort. We hypothesize that intrathecal morphine provides an equal opioid sparing effect as epidural analgesia with similar pain scores, without affecting length of hospital stay and time to fit for discharge.

## Methods

This study is a single-center retrospective study performed at the Rijnstate hospital in Arnhem, The Netherlands: a large teaching hospital and a center of referral for RARC-procedures. This manuscript adheres to the applicable STROBE guidelines. Prior to October 2020, all patients scheduled for a RARC-procedure received EDA, as recommended according to the Enhanced Recovery After Surgery (ERAS) guidelines for radical cystectomies [4]. In October 2020, intrathecal bupivacaine/morphine became the analgesic technique of choice, based on results demonstrated by other laparoscopic procedures in our hospital. No changes in clinical practice occurred during the study period.

The study was in accordance with the declaration of Helsinki and approved of by the hospital’s institutional review board on 21 January 2021 (Rijnstate Lokale Haalbaarheidscommissie, reference number 2020–1780). No written informed consent was required, since a retrospective study does not fall under the Medical Research involving Human Subjects Act (Dutch WMO-act). Per hospital policy, patients were informed that all clinical data can be used for retrospective analyses and patients were given the opportunity to oppose to the use of data. All patients were scheduled for RARC with or without pelvic lymph node dissection and with construction of a cutaneous uretero-ileostomy according to Bricker, in the time period of January 2019 until April 2021. Exclusion criteria were primary open surgery and the absence of neuraxial analgesia. The intrathecal cohort was determined by the time between change of practice and the time of evaluation (October 2020-April 2021). The epidural cohort was arbitrarily set on January 2019-October 2020, with the consideration of including sufficient patients, but without setting a too wide timeframe, so that unknown confounders could affect the research.

### Standard care

Patients were allowed enteral nutrition up to six hours prior to surgery and clear drinks up to two hours. No sedative premedication was administered and 2000 mg cefazolin and 500 mg metronidazole were administered intravenously as surgical site infection prophylaxis.

Both epidural and intrathecal analgesia were administered immediately prior to induction of anesthesia. For EDA, the patient was placed in the sitting position and the preference of the anesthesiologist determined the level and method of placement and the depth of catheter insertion. Typically, a level between thoracic 6 and 10 was preferred and the catheter was placed between 3 to 5 cm into the epidural space. A loading dose was administered at the anesthesiologist’s discretion. As maintenance regimen, a mixture of bupivacaine 1.25 mg/ml and sufentanil 1 mcg/ml was administered continuously between 6 and 12 ml/h. Intrathecal analgesia was administered with a 25- or 27-gauge needle (Pencan; Braun Melsungen AG, Melsungen, Germany), inserted at the lowest lumbar level possible until cerebrospinal fluid was obtained. Three to 5 ml of a pharmaceutically prepared mixture of bupivacaine 2.5 mg/ml and morphine 60 mcg/ml was administered, depending on the preference of the anesthesiologist.

Both groups received standardized general anesthesia, using 0.2 mcg/kg sufentanil, 2 mg/kg propofol and 0.6 mg/kg rocuronium. Subsequently their trachea was intubated, and the patient was positioned in the supine position. Anesthesia was maintained with continuous propofol or sevoflurane, depending on the preference of the anesthesiologist. In case of ventilator dyssynchrony or abdominal wall contraction, 10 mcg of intravenous rocuronium were given. When there was a greater than 10% increase in heart rate or blood pressure, 10 mcg of intravenous sufentanil were administered. Vasoactive medication (phenylephrine, ephedrine or norepinephrine) and fluid administration were given at the discretion of the attending anesthesiologist. Typically, a positive fluid balance of 1000–1500 ml was targeted. Prior to surgery, a transurethral catheter was inserted, the operative field was disinfected and covered in sterile drapes. Pneumoperitoneum up to a pressure of 15 mmHg was achieved by CO_2_-insufflation through a 12 mm camera trocar that was inserted via a periumbilical incision. Three 8 mm robotic trocars, a 15 mm and 5 mm trocar were then inserted before intra-abdominal pressure was decreased to 12 mmHg. The patient was placed in Trendelenburg position at 25 to 30 degrees and after the robot surgery system (Da Vinci Xi System, Intuitive Surgical, Sunnyvale, CA, USA) was docked, surgery started. In female patients, the urinary bladder, uterus, ovaries and ventral part of vaginal wall were resected. In male patients, urinary bladder and prostate were removed. After the radical cystectomy, pelvic lymphadenectomy was performed if indicated and an intracorporeal ileal conduit was constructed. The specimen bag was removed transvaginally in female patients or with a mini-laparotomy in male cases, after which the urostomy was created.

All patients received 1000 mg paracetamol at the start of surgery. Furthermore, patients received 1000 mg metamizole, 0.625 mg dehydrobenzperidol, 4 mg dexamethasone and/or 4 mg ondansetron at the discretion of the attending anesthesiologist.

After surgery, the patient was transferred to the Post Anesthesia Care Unit (PACU) for at least 30 min of observation. If patients required norepinephrine or there was an indication for intensive postoperative monitoring (such as, but not limited to, obstructive sleep apnea, severe heart failure or severe pulmonary compromise) they were admitted to the PACU, Medium Care or Intensive Care Unit for the first postoperative night. The unit of admission depended on availability.

In the epidural cohort, postoperative analgesia consisted of paracetamol 1000 mg four times daily and epidural bupivacaine 1.25 mg/ml and sufentanil 1 mcg/ml at a rate of 6–12 ml/hr. In the case of refractory postoperative pain, intravenous morphine was allowed in 2.5 mg increments. When patients with an epidural catheter were transferred to the ward, they were visited by nursing staff of the acute pain services daily to assure correct use of the epidural catheter and to answer questions from the ward if needed. At the third postoperative day, the epidural medication was stopped. If the pain was acceptable, the epidural catheter was removed and oxycodone 5–10 mg extended release was available up to three times daily if needed.

For the intrathecal morphine group, postoperative analgesia consisted of paracetamol 1000 mg four times daily, naproxen 250 mg three times daily and oxycodone 5–10 mg extended release up to three times daily if needed. Ondansetron 4 mg and haloperidol 1 mg was available for nausea and pruritus.

If pain was unresolved beyond this protocol, physicians could prescribe a Patient Controlled Analgesia (PCA)-pump if analgesia was unsatisfactory, or tramadol if the pain was deemed too mild for oxycodone.

### Data extraction and outcomes

Data were retrospectively extracted from the electronic patient data system (Hix 6.1, Chipsoft, Amsterdam, The Netherlands) and consisted of baseline characteristics (gender, weight, height, age, indication for surgery, medical history and ASA-classification), surgical characteristics (duration of surgery, estimated blood loss, type of urinary diversion and the need for conversion to an open procedure), anesthesia characteristics (type and specifics of analgesic method, intra-operative medication administration, fluid administration and dose and duration of vasopressors) and post-operative characteristics (admission to PACU, MCU or ICU and duration of admission, amount of fluid administration over the first two days and number of fluid challenges, urine production, blood transfusion, first day out of bed, first day of defecation, first day of enteral nutrition, length of hospital stay (LOS), time to fit for discharge (FFD) and re-admission to hospital within seven days after discharge). Postoperative pain characteristics consisted of the use of paracetamol and NSAIDs, duration of EDA, level of insertion, the dose of epidural medication, the reason for catheter removal, systemic morphine equivalent (ME) consumption over the first seven days, pain-scores on an 11-point Numeric Rating Scale (NRS; 0 is no pain, 10 is worst pain imaginable) over the first three days and occurrence of nausea, pruritus, hypotension (defined as Mean Arterial Pressure < 65 mmHg), respiratory depression (defined as a respiratory rate less than six breaths per minute), desaturation (defined as SpO2 < 90%) and motor blockade. The ME consumptions was converted with ratio of 1.0 for intravenous morphine, a ratio of 0.75 for oxycodone and 0.1 for tramadol. If a PCA-pump was provided to a patient, a ME consumption of 15 mg was assumed for that day, since the exact morphine consumption was not documented or retrievable. Pain scores on a NRS were measured once during every shift (day, evening and night) and when NRS was noted more than once, the highest value per shift was used to calculate a daily mean NRS. When NRS was higher than seven, it was registered as severe pain. The charts were reviewed for occurrence of complications and were classified according to the Clavien-Dindo classification. The following complications were defined: bradypnea, desaturation and infection (defined as a fever for which antibiotics were initiated and/or a rise in CRP together with a positive blood culture).

Fit for discharge (FFD) was defined as adequate analgesia with oral medication, hemodynamic stability, sufficient oral intake, passage of flatus and the capability of personal hygiene maintenance.

### Statistical analysis

Data were described as n(%) for categorical data or median (IQR)[range] for continuous data. All data were checked for normality by using a Kolmogorov–Smirnov test. Categorical data were analyzed with a Chi-squared test or a Fishers' exact test where appropriate. Continuous data were analyzed by using a Mann–Whitney-U-test. Correlations between continuous variables were tested with a Spearman Rho-test. Propensity Score Matching used a binary logistic regression in which the odds for inclusion in the epidural or intrathecal group was the dependent variable and gender, BMI, ASA-classification and age were the independent variables. *P* < 0.05 was considered statistically significant. SPSS 26.0 (IBM, Armonk, NY, USA) was used for the analysis.

## Results

One hundred and seventy-five patients were screened, 22 of whom were excluded because no consent was obtained for a neuraxial technique preoperatively (*n* = 11), a neuraxial technique was not initiated by the attending anesthesiologist (*n* = 7), it was initiated but not possible (*n* = 3, two of which were EDA) and in one patient the surgery was prematurely ended because of metastatic disease (*n* = 1). One hundred and fifty-three patients were analyzed, 114 in the epidural group and 39 in the ITM group. Baseline patient’s characteristics, together with the neuraxial anesthesia characteristics, are displayed in Table 1. In eight patients (five in the epidural group, three in the ITM group), no bladder extraction was carried out because of progressive invasive disease. Furthermore, two patients received a neobladder instead of a Bricker derivation (both in the epidural group). Since these patients did receive a urinary diversion and their data did not show great outliers that would cause bias, they were included in the analysis.

**Table 1:** Baseline Characteristics. The epidural cohort is demonstrated in full and as matched cohort according to the propensity to receive intrathecal analgesia. Continuous data is presented as median (IQR)[range]. ASA: American Society of Anesthesiology, BMI: Body Mass Index

	**Intrathecal analgesia (*n* = 39)**	**Epidural analgesia (*n* = 114)**	***p***	**Matched Epidural Cohort (*n* = 39)**	***p***
Age (years)	71 (65–77)[49–83]	70 (65–78)[42–88]	0.969	69 (65–77)[42–85]	0.656
Male, n (%)	24 (62)	79 (69)	0.430	23 (59)	1.000
BMI (kg/m^2^)	24.3 (22.5–29.0)[18.8–33.7]	26.1 (23.9–29.7)[17.1–37.3]	0.101	24.8 (23.0–27.5)[17.1–35.5]	0.795
ASA classification (1/2/3/4), n (%)	0/25/12/2 (0/64/31/5)	10/58/42/4 (9/51/37/4)	0.183	0/24/13/2 (0/61/33/5)	0.970
Malignancy as indication, n (%)	38 (97)	112 (98)	1.000	38 (97)	1.000
Previous abdominal surgery, n (%)	6 (15)	29 (25)	0.270	14 (36)	0.068
Pre-operative opioid use, n (%)	6 (15)	9 (8)	0.210	2 (5)	0.186
Mode of extraction, n (%)			0.631		0.539
- Vaginal (in total group/in women)	13 (33/80)	32 (29/91)		15 (38/100)	
- Trocar opening	23 (59)	76 (67)		23 (59)	
- Only diversion	3 (8)	6 (5)		1 (3)	
Level of epidural catheter placement, n (%)					
- Th4-Th7		4 (4)		0 (0)	
- Th7-Th12		87 (76)		29 (74)	
- Th12-L5		17 (15)		8 (20)	
- Not recorded		6 (5)		2 (5)	
Epidural opioids, n (%)		112 (98)		39 (100)	
Epidural continuous dose (mL/h)		6 (6–8)[4–12]		6 (6–8)[4–12]	
Day of epidural catheter removal		3 (2–3)[0–4]		3 (2–3)[0–4]	
Cause of removal					
-Elective		79 (69)		26 (67)	
- Dislocation		25 (22)		11 (28)	
- Complications		10 (9)		2 (5)	
Intrathecal morphine dose (µg)	240 (180–210)[120–300]				

The epidural catheters remained in situ for over a median of three (2–3)[0–4] days and were removed because they were no longer needed (*n* = 79, 69%), the catheter dislocated (*n* = 25, 22%) or the side effects such as hypotension or severe motor block prevented further use of the epidural catheter (*n* = 10, 9%). The median dose of intrathecal morphine was 240 µg (180–210)[120–300]. Table 2 demonstrates intraoperative variables. Surgical times were longer in the epidural group (320 min (278–365)[159–602] vs 280 min (249–330)[210–483], *p* = 0.004) and the intrathecal group received more NSAIDs (*n* = 27 (24%) vs *n* = 30 (77%), *p* < 0.001) and dehydrobenzperidol *n* = 4 (4%) vs *n* = 6 (15%), *p* = 0.018). Eight patients (7%) in the epidural group and four (10%) in the intrathecal group received a PCA-pump on the ward (*p* = 0.500).

**Table 2:** Intra-operative variables. Continuous data is presented as median (IQR)[range]. Min = minutes, NSAID = Non-Steroidal Anti Inflammatory Drugs

	**Intrathecal analgesia (*n* = 39)**	**Epidural analgesia (*n* = 114)**	***p***	**Matched Epidural cohort**	***p***
Duration of surgery (min)	280 (249–330)[210–483]	320 (276–375)[159–602]	0.004	299 (272–352)[210–450]	0.680
Estimated blood loss (ml)	150 (100–300)[15–500]	150 (50–250)[0–1000]	0.937	150 (75–250)[25–350]	0.875
Sevoflurane, n (%)	36 (92)	105 (92)	1.000	36 (92)	1.000
Paracetamol, n (%)	39 (100)	108 (95)	0.339	38 (97)	1.000
NSAID, n (%)	30 (77)	27 (24)	< .001	11 (28)	< .001
Cumulative rocuronium dose (mg)	116 (90–130)[39–215]	127 (77–168)[52–620]	0.218	101 (77–155)[52–238]	0.889
Corticosteroids, n (%)	34 (87)	105 (92)	0.316	37 (95)	0.431
Ondansetron, n (%)	29 (74)	81 (71)	0.675	27 (69)	0.472
Dehydrobenzperidol, n (%)	6 (15)	4 (4)	0.018	2 (5)	0.262
Crystalloid administration (ml)	1156 (951–1568)[162–2572]	1292 (977–1630)[258–3349]	0.465	1295 (998–1571)[686–2513]	0.651

### Pain scores

The pain scores on the first two postoperative days were slightly higher in the intrathecal analgesia group (Table 3). The number of patients reporting severe pain (NRS > 7) was similar in both groups (EDA vs intrathecal analgesia, on POD0: *n* = 7 (6%) vs *n* = 0 (0%), *p* = 0.191; POD1: *n* = 9 (9%) vs *n* = 3 (9%), *p* = 1.000; POD2: *n* = 5 (5%) vs *n* = 2 (7%), *p* = 0.661 and POD3: *n* = 6 (6%) vs *n* = 0 (0%), *p* = 0.335). Total postoperative ME consumption was similar over the seven postoperative days, resulting in a total postoperative ME consumption of 15 mg (5–35)[0–148) in the epidural group versus 11 mg (0–35)[0–148] in the intrathecal group (*p* = 0.167). Patients in the intrathecal group consumed more ME on the day of surgery and the two days after, while the epidural group consumed more ME on POD three to seven (Table 3).

**Table 3:** Postoperative pain scores and morphine equivalent consumption. Continuous data is presented as median (IQR)[range]. NRS: numeric rating scale. ME: morphine equivalent

	**Intrathecal analgesia (*n* = 39)**	**Epidural analgesia (*n* = 114)**	***P***	**Matched Epidural analgesia**	***p***
Mean postoperative pain scores					
- Day 0 (NRS)	1 (0–3)[0–5]	0 (0–2)[0–8]	0.050	0 (0–2) [0–8]	0.164
- Day 1 (NRS)	3 (1–4)[0–7]	2 (1–3)[0–8]	0.058	2 (1–3) [0–8]	0.106
- Day 2 (NRS)	3 (2–4)[0–7]	2 (0–3)[0–8]	0.010	2 (0–4) [0–6]	0.037
- Day 3 (NRS)	3 (2–4)[0–6]	2 (1–4)[0–10]	0.676	2 (1–4) [0–7]	0.575
Morphine Equivalent Consumption					
- Intraoperatively (mg)	0 (0–0)[0–10.5]	0 (0–0)[0–14]	0.046	0 (0–0)[0–14]	0.080
- Day 0 (mg)	0 (0–0)[0–35]	0 (0–0)[0–15]	< .001	0 (0–0)[0–15]	0.031
- Day 1 (mg)	2.5 (0–10)[0–35]	0 (0–0)[0–25]	< .001	0 (0–5)[0–20]	0.011
- Day 2 (mg)	0 (0–10)[0–30]	0 (0–5)[0–20]	0.049	0 (0–3.5)[0–15]	0.098
- Day 3 (mg)	0 (0–5)[0–37.5]	5 (0–10)[0–20]	0.006	5 (0–10)[0–17.5]	0.056
- Day 4 (mg)	0 (0–0)[0–20]	0 (0–10)[0–25]	0.017	0 (0–10)[0–25]	0.040
- Day 5 (mg)	0 (0–0)[0–30]	0 (0–5)[0–17.5]	0.056	0 (0–10)[0–17.5]	0.067
- Day 6 (mg)	0 (0–0)[0–15]	0 (0–5)[0–32.5]	0.014	0 (0–5)[0–32.5]	0.007
- Day 7 (mg)	0 (0–0)[0–15]	0 (0–0)[0–30]	0.042	0 (0–5)[0–30]	0.016

### Recovery

There were no differences in time to first mobilization (day 1 (1–1)[0–5) vs day 1 (1–1)[0–2], *p* = 0.389), time to first enteral nutrition (day zero (0–1)[0–2] vs day zero (0–1)[0–1], *p* = 0.360) and time to first bowel movement (day three (2–4)[0–10] vs day three (2–4)[2–12], *p* = 0.256). Both the actual LOS and days until FFD were higher in the epidural group (respectively seven days (5–9)[4–42] vs six days (5–7)[4–38], *p* = 0.006, and five days (4–8)[3–30]) vs five days (4–6)[3–34], *p* = 0.018).

### Complications

We found no difference in the postoperative course between the two groups (see Table 4). The PACU/ICU/MCU admission rate was 61 (54%) in the EDA group and 21 (54%) in the ITM group (*p* = 0.707). Fifty-three patients in the EDA group (46%) versus fourteen in the ITM group (36%) required norepinephrine after surgery (*p* = 0.348). Complications based on the Clavien-Dindo classification were similar. Furthermore, no difference was found between the EDA and the ITM group for pruritus (*n* = 11 (10%) vs *n* = 1 (3%), *p* = 0.297), nausea (*n* = 53 (46%) vs *n* = 20 (51%), *p* = 0.580), motor block (*n* = 3 (3%) vs *n* = 0 (0%), *p* = 0.572), bradypnea (*n* = 2 (2%) vs *n* = 0 (0%), *p* = 1.000), and desaturation (*n* = 19 (17%) vs *n* = 6 (16%), *p* = 1.000). Eight people in the EDA group (7%) required re-admission after initial discharge, versus none in the ITM group (*p* = 0.203).

**Table 4:** Post-operative outcomes and adverse events. Infection was defined as a fever and/or rise in CRP and positive culture. Continuous data is presented as median (IQR)[range]

	**Intrathecal analgesia (*n* = 39)**	**Epidural analgesia (*n* = 114)**	***P***	**Matched Epidural cohort (n = 39)**	***P***
Duration of postoperative norepinephrine administration (h)	6 (4.5–8)[2–24.5)	6.5 (5–9)[0–60)	0.093	7.5 (5–9)[0–60]	0.064
Urine production in the first two days (ml)	4057 (2967–5656)[1818–8480]	3735 (2940–4750)[514–12.420]	0.268	3817 (2761–5275)[1440–6946]	0.505
Fluid Challenges in the first two days (ml)	500 (500–875)[250–1000]	500 (500–1250)[500–2500]	0.266	500 (500–1500)[500–2500]	0.200
Clavien-Dindo classification, n (%)			0.331		0.237
- 0	8 (21)	27 (24)		6 (15)	
- 1	19 (49)	34 (30)		12 (31)	
- 2	10 (26)	41 (36)		15 (38)	
- 3	1 (3)	9 (8)		5 (13)	
- 4	1 (3)	2 (2)		1 (3)	
- 5	0 (0)	1 (1)		0 (0)	
Infection, n (%)	5 (13)	30 (26)	0.121	9 (23)	0.377
Pneumonia, n (%)	2 (5)	2 (2)	0.601	1 (3)	1.000
Anastomotic leakage, n (%)	0 (0)	2 (2)	1.000	0 (0)	N/A
Ileus, n (%)	7 (18)	20 (18)	0.808	7 (18)	1.000
Delirium, n (%)	2 (5)	6 (5)	1.000	2 (5)	1.000
Transfusion, n (%)	5 (13)	13 (11)	0.768	5 (13)	1.000
Parenteral nutrition, n (%)	4 (10)	19 (17)	0.596	7 (18)	1.000

### Propensity matched cohort

An epidural cohort was matched with regard to age, gender, ASA-classification and BMI with the intrathecal cohort. The major outcomes are reported in the tables, showing minor differences with the entire epidural cohort. In addition, the total postoperative opioid consumption on the ward was 15 mg (5–40)[0–147.5) in the matched epidural cohort and did not differ from the ITM-group (*p* = 0.222). Compared to the ITM-group, the actual LOS was 7 days (6–9)[5–30], *p* = 0.006 and the time until FFD was 6 days (4–9)[3–29], *p* = 0.016) in the matched epidural cohort.

## Discussion

The results of this study demonstrated that postoperative pain scores and ME consumption were slightly higher in the ITM group on postoperative day zero to two. Overall, ME-consumption was comparable in the ITM and the EDA group, because the EDA group received more ME on postoperative days 3, 4, 6 and 7. Furthermore, there was no difference between the two groups in terms of time to mobilization and postoperative complications. The EDA group did take longer to become fit for discharge and their length of hospital stay was also longer than that of the ITM group, but this was a small difference. These findings were substantiated with similar outcomes in a Propensity Matched Cohort. From our findings, it could be concluded that the opioid sparing effect of ITM is of the same size as that of epidural analgesia.

Compared to another study involving RARC without locoregional or neuraxial analgesia, both the EDA and the ITM group in our study showed an important reduction in opioid consumption [5]. Noteworthy is the rise in ME consumption in the EDA group after two days, whereas the ITM group showed a decrease. This divergent phenomenon might be a sign of rebound pain after removal of the epidural catheter, while the patients in the ITM group are already better adjusted to the postoperative pain. However, the adjustment to pain may seem contradictory, given that previous models show that pain sensitizes patients for pain rather than desensitizing them [10]. It could also be hypothesized that intense analgesia provided by EDA leads to a diminished action of the inhibiting descending pain pathways. Alternatively, because confounding factors cannot be excluded in retrospective research, perhaps an unidentified confounder in postoperative care may be the cause of this finding.

The length of hospital stay was only one day longer in the EDA group, and the difference in time until FFD was even less. Since there was no difference in the demographics of both patient groups and the difference remained in the Propensity Matched Cohort, it seems unlikely that factors like age, gender or ASA-classification have contributed to the prolonged length of stay. Late removal of the epidural catheter also does not seem to delay hospital discharge, because the mean time until FFD is five days in the EDA group and the epidural catheters were usually removed on day three. Other indices of recovery, such as time to mobilization and time to enteral nutrition, also did not differ between the two groups. We therefore believe that the prolonged stay in the EDA group is caused by a gradual change of practice towards earlier discharge, since the admission of the EDA cohort was longer ago than that of the ITM group. It is not likely that this was affected by the learning curve of the surgeons, as they all had reached the plateau phase of the learning curve for RARC [11].

No significant difference in postoperative complications between the two groups was found when looking at the Clavien-Dindo classification overall. Furthermore, there was also no difference in major complications, defined as a Clavien-Dindo score of 3 and higher. Intrathecal morphine did not lead to a higher incidence of nausea during the first two postoperative days, presumably because adequate intra-operative prophylaxis with ondansetron, steroids and dehydrobenzperidol was given. Moreover, there was no difference in bradypnea, pruritus or desaturation, which are known side effects of ITM [12]. Previous research has stated that the low dosage of intrathecal morphine is unlikely to cause respiratory problems and the current study seems to confirm this [9, 13]. However, our conclusions are based on measurements that were commonly made only once per shift and registration of pruritus was also partly dependent on whether patients deemed it severe enough to report it. Because of the low frequence of measurements and the partially patient dependent registration, a certain degree of bradypnea, desaturations and pruritus might have been missed. However, if this is true, it is debatable if such an event would have been clinically meaningful.

Limitations of our study are in a major part caused by the retrospective design. A Propensity Score Matching was added, but only as a consolidation of the conventional analysis, since this Propensity Score Matching may still be subject to unknown confounders. Missing data was important with regard to the actual amount of PCA administered opioids, which could not be retrieved. However, only 8% of the patients received a PCA-pump, equally distributed over both cohorts, so the lack of data caused by this suboptimal registration is equally distributed as well. Another limitation of the retrospective design is the registration of infections: in some cases, antibiotics might have been initiated on fever alone. Since this is a non-specific indicator for infections, the actual incidence of postoperative infections may have been overestimated. However, since this happened in the EDA group as well as the ITM group, we do not expect this to have caused bias. Lastly, the great difference in size of the EDA group compared to the ITM group is also a limitation. The Propensity Score Matched cohort was added to correct for the difference in group size and four baseline characteristics, and the findings remained. The timing of the current analysis was determined to evaluate the change of practice after six months for clinical reasons. This led to a lower number of patients in the ITM group, but still sufficient to show important differences. Still, a larger cohort might have given even more insight into the effects of ITM.

## Conclusions

In conclusion, our results show that both epidural analgesia and intrathecal morphine lead to adequate analgesia for patients who undergo RARC with Bricker derivation. A prospective study may be performed to investigate if ITM may be preferred over epidural catheters, because of its less invasive and simpler technique. Until then, intrathecal morphine may be a suitable alternative, when epidural analgesia is not feasible.

## Data Availability

Data is available upon reasonable request by the corresponding author (M.V. Koning).
